# Loop-mediated isothermal amplification (LAMP): An advanced molecular point-of-care technique for the detection of *Leishmania* infection

**DOI:** 10.1371/journal.pntd.0007698

**Published:** 2019-11-07

**Authors:** Chukwunonso O. Nzelu, Hirotomo Kato, Nathan C. Peters

**Affiliations:** 1 Snyder Institute for Chronic Diseases, Departments of Microbiology, Immunology, and Infectious Diseases, Cumming School of Medicine and Comparative Biology and Experimental Medicine, Faculty of Veterinary Medicine, University of Calgary, Calgary, Canada; 2 Division of Medical Zoology, Department of Infection and Immunity, Jichi Medical University, Tochigi, Japan; Pasteur Institute of Iran, ISLAMIC REPUBLIC OF IRAN

## Abstract

Leishmaniasis, caused by protozoan parasites of the *Leishmania* genus, represents an important health problem in many regions of the world. Lack of effective point-of-care (POC) diagnostic tests applicable in resources-limited endemic areas is a critical barrier to effective treatment and control of leishmaniasis. The development of the loop-mediated isothermal amplification (LAMP) assay has provided a new tool towards the development of a POC diagnostic test based on the amplification of pathogen DNA. LAMP does not require a thermocycler, is relatively inexpensive, and is simple to perform with high amplification sensitivity and specificity. In this review, we discuss the current technical developments, applications, diagnostic performance, challenges, and future of LAMP for molecular diagnosis and surveillance of *Leishmania* parasites. Studies employing the LAMP assay to diagnose human leishmaniasis have reported sensitivities of 80% to 100% and specificities of 94% to 100%. These observations suggest that LAMP offers a good molecular POC technique for the diagnosis of leishmaniasis and is also readily applicable to screening at-risk populations and vector sand flies for *Leishmania* infection in endemic areas.

## Author summary

Developing sensitive point-of-care diagnostic tests is vital for enhancing *Leishmania*-infection control programs and treatment. This review provides information on the development of the loop-mediated isothermal amplification (LAMP) diagnostic test and highlights recent advances in the field of molecular diagnosis of leishmaniasis and the needs for future research. Furthermore, we elaborate of the future potential of LAMP as a rapid point-of-care (in-clinic and in the field) test for diagnosis and entomological monitoring of *Leishmania* infection, including evaluation of control programs in *Leishmania*-endemic areas.

## Introduction

Leishmaniasis is a diverse spectrum of clinical syndromes caused by more than 20 species of the obligate intracellular protozoa parasites of the genus *Leishmania* and is transmitted by the bite of an infected female sand fly. Leishmaniasis is one of the most neglected and poverty-related tropical diseases in the world [1]. The disease is endemic in 98 countries, affecting 12 million people worldwide, with an estimated 350 million people at risk of infection [2]. Unfortunately, in most countries, the incidence of disease is often underestimated largely due to unrecognized cases, lack of access to healthcare, or under-reporting [3]. Different species of *Leishmania* cause disease in both humans and animals, and *Leishmania* is often referred to as a zoonosis. After deposition into the skin, *Leishmania* can give rise to mild and atypical types of cutaneous leishmaniasis (CL), a destructive mucocutaneous leishmaniasis (MCL), and deadly systemic visceral leishmaniasis (VL). The clinical manifestations of disease are largely associated with the infecting strain of the parasite. Considering leishmaniasis is an emerging and uncontrolled disease in some regions and is increasing in some endemic areas, timely diagnosis and treatment of patients is of paramount importance to contain the disease.

Currently available diagnostic tools for leishmaniasis can be divided into three groups: parasitological, serological, and molecular diagnostics, each of them coming with advantages and disadvantages. The traditional parasitological methods, which include microscopy and parasite culturing, remain the diagnostic gold standard. Though technically specific, it suffers from low sensitivity, and only a handful of health centers in the field are able to culture parasites. Serological methods with comparable sensitivity to parasitological methods are available in the form of enzyme-linked immunosorbent assay (ELISA) and rapid diagnostic tests (RDTs) based on rK39 [4, 5], immunofluorescence antibody test (IFAT), western blotting, and direct agglutination test (DAT) [6, 7]. However, serology-based diagnostic techniques also have some disadvantages, such as cross-reactivity and false-positive results [4, 6, 7]. Molecular methods provide an effective alternative to the aforementioned methods and are of greater sensitivity and specificity [6, 7]. The presence of *Leishmania* DNA as a molecular biomarker for infection can be effectively employed in both human and other mammalian hosts. Human clinical samples, such as whole blood, urine, bone marrow, lymph nodes, serum, buffy coat [6], and cutaneous lesion aspirates or scrapings [8, 9], have reliably been used to detect the presence of parasitic DNA. Despite the apparent availability of effective diagnostics tests, resource limitations in endemic countries or a lack of experience among physicians and laboratory technicians in nonendemic countries leads to either no diagnosis or delays and inaccurate diagnosis. Consequently, a sensitive and specific molecular diagnostic method is required in both endemic and nonendemic regions. One of the most basic and widely used molecular diagnostic techniques is polymerase chain reaction (PCR) detection of *Leishmania* DNA [6, 7]. Detection of pathogen DNA directly from clinical samples has permitted more analytically sensitive diagnosis of infection. However, the technique requires expensive equipment, DNA purification, a long time to diagnosis, and a lack of field applicability, thereby preventing the use of PCR-based diagnostics in resource-limited, disease-endemic settings. To overcome PCR limitations, in the year 2000, a novel molecular technique, the loop-mediated isothermal amplification (LAMP), was developed as a field-friendly and cost-effective diagnostic tool [10], and it appears to be a feasible molecular diagnostic tool for both endemic and nonendemic regions. LAMP has the advantage of no major capital equipment requirement, simplified DNA extraction methods like boil and spin, naked eye detection of amplification, and the use of dry reagents, including the polymerase enzyme.

**Fig 1:**
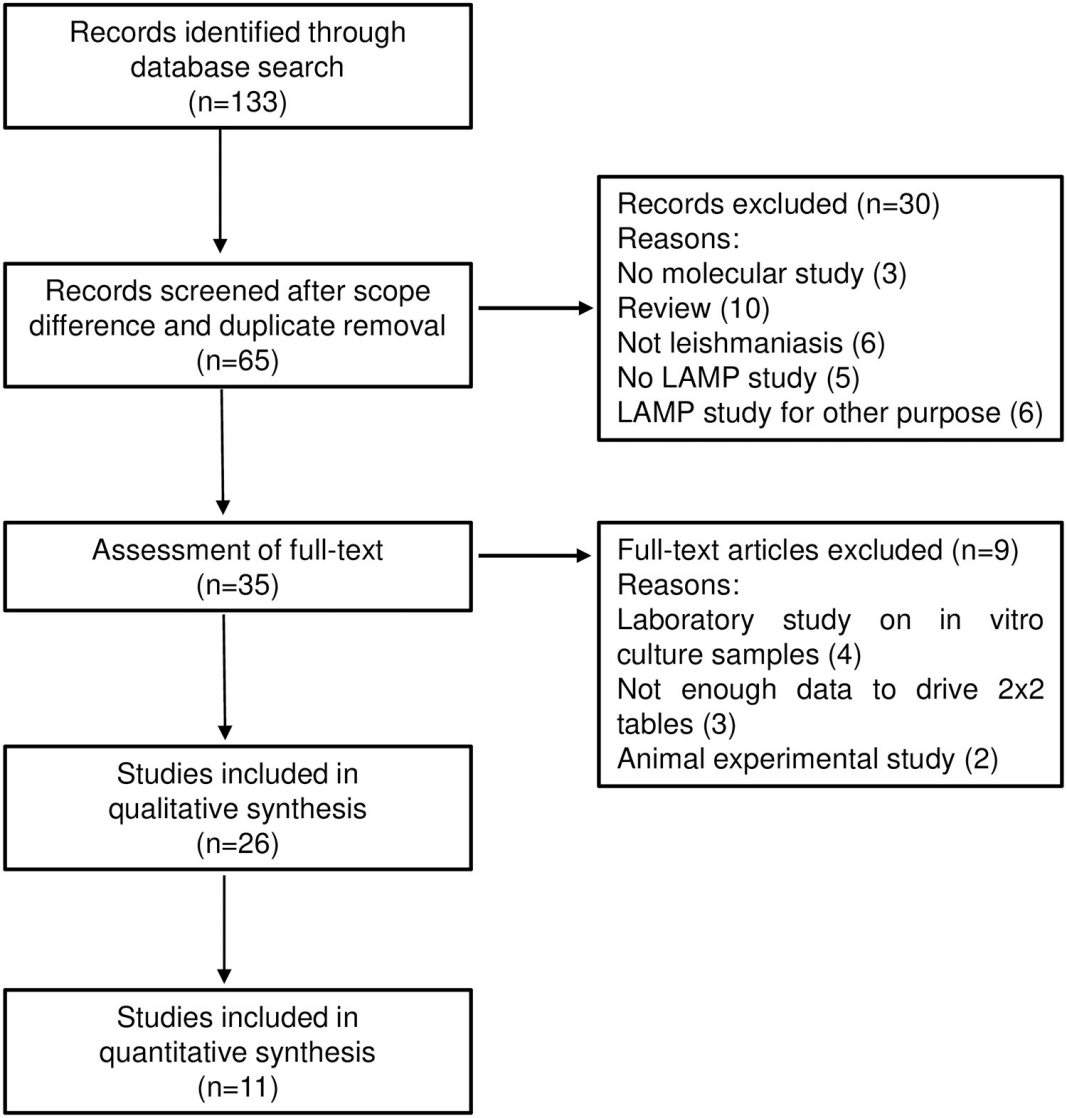
Flowchart of the studies identified, screened, and included in this review.

Several studies have shown the utility of the LAMP technique as a useful tool for rapid detection of pathogenic agents (bacteria, parasites, and viruses) of infectious diseases [9, 11–15]. LAMP diagnostic kits have also been developed by the Eiken Chemical Co. (Japan) for human African trypanosomiasis (HAT) [16], tuberculosis [17], malaria [18, 19], and leishmaniasis [20, 21]; and recently the LAMP diagnostic kit for tuberculosis has been endorsed by the World Health Organization [22]. Meridian Biosciences (Cincinnati, OH) has also developed a centrifugation-free assay called *illumi*gene for malaria genus-level detection [23]. Since the advent of LAMP as a simple and robust nucleic acid amplification test (NAAT), different studies have shown a number of prototype LAMP assays in the field of leishmaniasis. Here we present a review, current status, diagnostic performance, and perspectives of molecular-based LAMP technique for leishmaniasis, as well as its prospects as a xenomonitoring/surveillance tool in endemic areas. Box 1 shows the search strategy and selection criteria used in this review. The flow of included studies is graphically presented in Fig 1.

**Box 1. Information sources, search strategies, and study selection.**

We searched for articles in PubMed, Embase, Web of Science, Google Scholar, and Scopus, with the following keywords: *Leishmania* LAMP assay; cutaneous leishmaniasis; molecular diagnosis; PCR and LAMP; *Leishmania* diagnosis; colorimetric dye; parasitic DNA; leishmaniasis; mucocutaneous leishmaniasis; visceral leishmaniasis; post kala-azar dermal leishmaniasis; canine leishmaniasis; and sand fly, surveillance, xenomonitoring, and other related words.

The eligibility criteria included the following: original studies evaluating LAMP test; clinical cutaneous leishmaniasis, visceral leishmaniasis, post kala-azar dermal leishmaniasis in human, canine leishmaniasis, and *Leishmania* detection in sand flies as respective target conditions; adequate reference classification; and absolute numbers of true-positive, true-negative, false-positive, and false-negative observations derivable from the data presented in the diagnosis of human leishmaniasis. Commercial and laboratory developed tests were eligible. LAMP test accuracy were summarized as sensitivity and specificity with 95% confidence intervals. Analysis was performed using *Review Manager* (*RevMan*) *Version 5*.*3*. *Copenhagen*: *The Nordic Cochrane Centre*, *The Cochrane Collaboration*, *2014*.

## LAMP technique: Its developments and features

**Fig 2:**
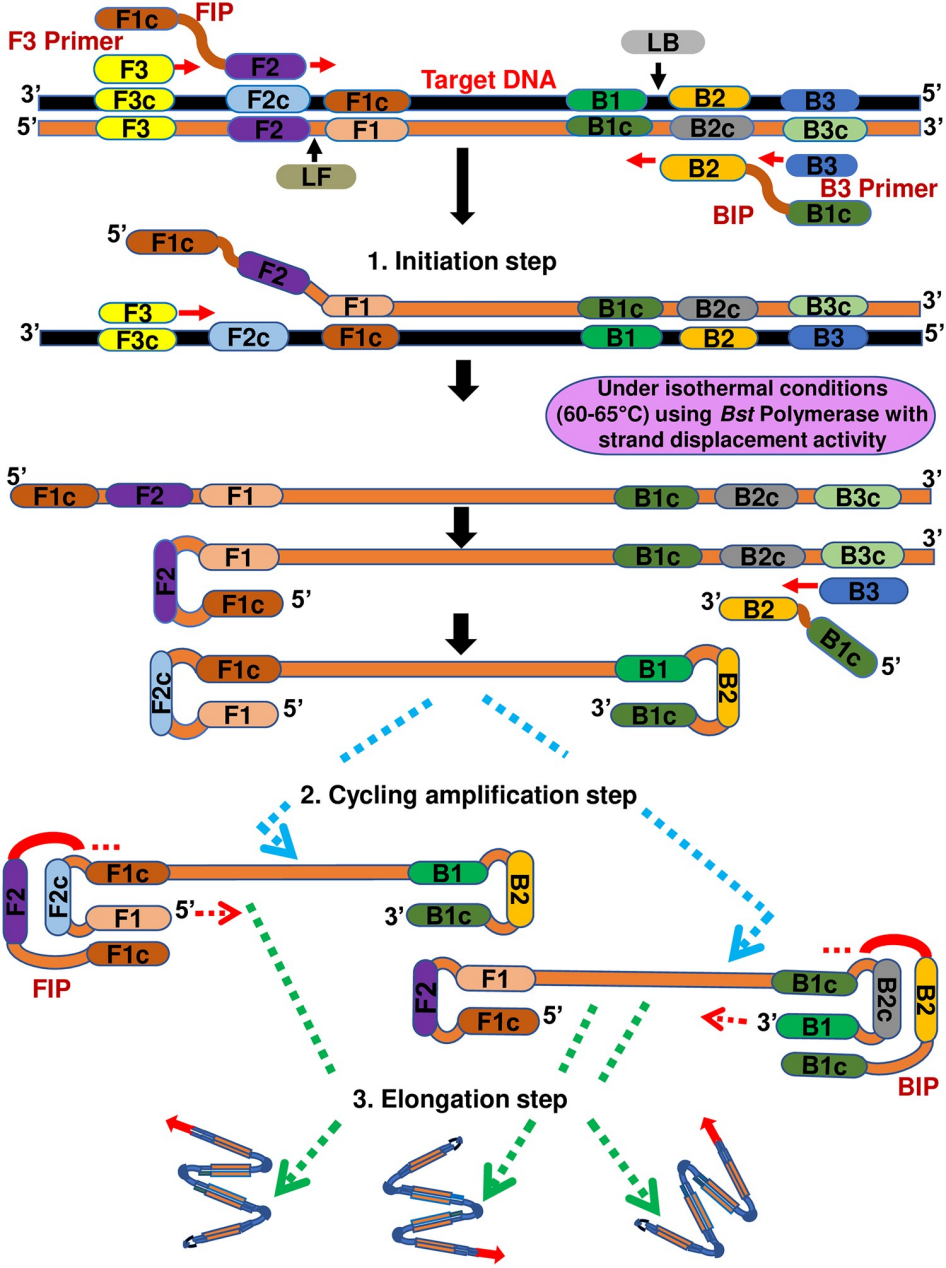
Schematic representation of molecular mechanism of LAMP (three major steps in the LAMP reaction) and localization of the LAMP primers on target DNA sequence.

A recent advance in molecular (nucleic acid-based) diagnostics has been the development of LAMP [10], among others like nucleic acid sequence-based amplification (NASBA) and recombinase polymerase amplification (RPA) [24, 25]. The LAMP technique employs a *Bacillus stearothermophilus* (*Bst*) DNA polymerase—which has both polymerase and reverse transcriptase activity—and a set of four primers (two inner primers, with typical length of approximately 40–42 bp and two outer primers, with typical length approximately 17–20 bp) that recognize six distinct sequences of the target DNA, which makes them highly specific to the target. Occasionally, the addition of two extra primers (loop forward primer and loop backward primer, with a typical length of approximately 20 bp) referred to as loop primers accelerates the amplification reaction, thereby decreasing the reaction time required [26]. The design of the LAMP primers is easy and can readily be done through a user-friendly online platform: Primer Explorer V4 software (http://primerexplorer.jp/e) running in java Runtime environment, a product of Eiken Chemical Co. LAMP has the capacity to amplify a few copies of DNA to 10^9^ in <60 min with high efficiency [10]. The mechanism behind the LAMP reaction involves three major steps: initiation, cycling amplification, and elongation (Fig 2). Typically, the reaction begins with the binding of the inner primers containing sequences of the sense and antisense strands of the target DNA, and this is followed by strand displacement DNA synthesis by the outer primers (initial step). Subsequently, the cyclical amplification step and elongation occur [10]. One good feature of LAMP is the auto strand displacement properties of *Bst* polymerase, which enables amplification reaction using heating block or normal water bath maintained at a specific temperature without the use of expensive thermal cyclers. The appearance of magnesium pyrophosphate precipitate (a by-product of DNA amplification) provides a positive indicator of the target DNA amplification. Real-time turbidimetry facilitates the quantification of the template DNA in the reaction and allows the analysis of minute quantities of DNA. Furthermore, LAMP amplicon can be analyzed using agarose gel electrophoresis and/or simple colorimetric naked eye visualization closed detection systems [27] and real-time fluorimetry [21, 28]. Therefore, the major advantage of LAMP is its application in a field or resource-limited setting.

## Application of LAMP technique to leishmaniasis

### CL

CL is the most prevalent clinical form of leishmaniasis worldwide, characterized by the presence of ulcerative lesions leading to disfiguring and/or incapacitating scars [2]. CL is endemic in the tropics and neotropics and in more than 70 countries worldwide, and 90% of cases occur in seven countries: Afghanistan, Algeria, Brazil, Iran, Peru, Saudi Arabia, and Syria [29]. CL is most commonly associated with *Leishmania* (*Leishmania*) *major*, *L*. (*L*.) *tropica*, and *L*. (*L*.) *aethiopica* species in the Old World and multiple species of *Leishmania*—*L*. (*L*.) *mexicana*, *L*. (*L*.) *amazonensis*, *L*. (*Viannia*) *braziliensis*, *L*. (*V*.) *guyanensis*, *L*. (*V*.) *panamensis*, and *L*. (*V*.) *peruviana*—in the New World [30]. In some regions, two or more species are often sympatric [31]. Species prevalent in the Old World in most cases cause self-limiting ulcers, while the New World species are known to cause a syndrome called American tegumentary leishmaniasis—comprised of CL and a variety of other manifestations, like MCL—and a diffuse and disseminated cutaneous leishmaniasis (DCL or diffuse-CL) [32].

Because the clinical spectrum of CL is broad and can be misdiagnosed as other skin conditions such as cutaneous mycoses, leprosy, keloid, lupus vulgaris, or sarcoidosis [33], a differential diagnosis can be very important for treatment. While microscopy and culture remain the gold standard for CL diagnosis, the sensitivity is variable and time consuming, demanding technical expertise and significant infrastructure. Serology-based method is not very useful in CL diagnosis because of undetectable or low antibodies titers. Many molecular diagnostic methods that allow the use of less invasive sampling with better sensitivity and specificity have been developed extensively for CL diagnosis. Particularly, PCR-technique targeting different gene sequences have been employed over the last decades for CL diagnosis [34, 35]. PCR platforms for CL show specificity (84%–100%) and sensitivity (90%–98%) [34, 36].

**Fig 3:**
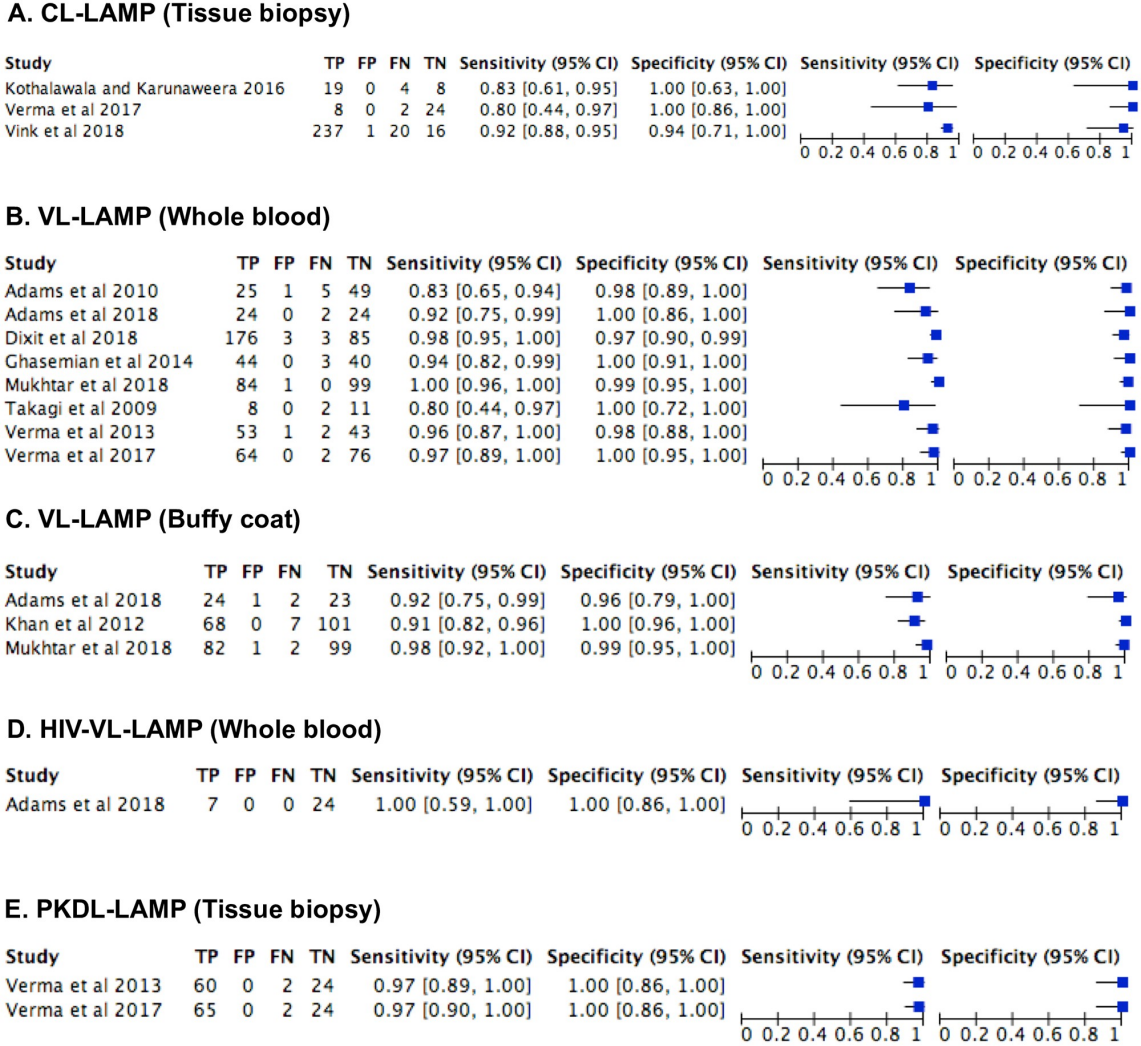
Reported LAMP diagnostic accuracy by study and test with forest plots. (A) Cutaneous leishmaniasis (CL)-LAMP on tissue biopsy (B) Visceral leishmaniasis (VL)-LAMP on whole blood. (C) VL-LAMP on buffy coat. (D) HIV-VL co-infection-LAMP on whole blood. (E) Post kala-azar dermal leishmaniasis (PKDL)-LAMP on tissue biopsy. FN, False-negative; FP, False-positive; TN: True-negative; TP, True-positive.

The first LAMP test for CL on skin biopsy was a generic, reverse transcriptase (RT-LAMP), targeting the conserved region of the *Leishmania* 18S ribosomal RNA (rRNA) gene with infection detection limit of 10 and 100 parasites/ml [37]. Different studies have recently developed this technique for various applications, especially those based on the *Leishmania* 18S rRNA and minicircle kinetoplast DNA (kDNA) genes in CL endemic countries (Table 1). The choice of 18S rRNA gene in most pan-*Leishmania* LAMP assays was due to its high conservation across *Leishmania* species, while the kDNA gene has been proven to yield high sensitivity against other genes in comparative studies due to its high copy number [38, 39]. More recently, a LAMP assay targeting the cysteine protease B (cpb) gene has also been used for diagnosis of CL cases in Tunisia [40]. A comparative study of pan-*Leishmania* LAMP assays employing primers targeting *Leishmania* 18S rRNA and histone genes, respectively, using purified DNAs of CL-causing species—*L*. (*L*.) *major*, *L*. (*L*.) *tropica*, *L*. (*L*.) *mexicana*, *L*. (*V*.) *braziliensis*, *L*. (*V*.) *guyanensis*, *L*. (*V*.) *panamensis*—revealed a similar limit of detection at 0.01 parasite/μl for both genes, but the histone primer was found to be incapable of amplifying all *L*. (*V*.) *guyanensis* and *L*. (*V*.) *braziliensis* strains tested, indicating low sequence homology to some strains [39]. Studies have shown the usefulness of patient saliva [41] and a direct boil-LAMP method [42] in the diagnosis of CL. The boil method was found not to compromise *Leishmania* detection sensitivity of the LAMP assays [42, 43]. Furthermore, a Flinders Technology Associates (FTA)-LAMP for CL with detection sensitivity as low as 0.01 parasites/μl was demonstrated with clinical tissue spotted on FTA cards obtained from patients in Peru [9]. The established LAMP assay showed the usefulness of FTA cards as a direct sampling tool for diagnosis of CL and was comparable to using purified DNA as a template. The reported FTA-LAMP is a further improvement in the application of LAMP in that it circumvents the need for liquid handling during sample collection and transportation, as well as refrigerant/cold storage. In spite of the fact that only a few human CL-LAMP studies were identified in our review of available literature, the diagnostic accuracy of LAMP testing for CL revealed sensitivity of 80%–92% and specificity of 94%–100% on tissue biopsies in three studies [44–46] (Fig 3A). Overall, these reports support a potential role of LAMP as a reliable point-of-care (POC) diagnostic test for CL, which will be widely applicable in both endemic and nonendemic regions.

**Table 1:** Overview of LAMP assays for diagnosis of human and canine leishmaniases reported in previous studies.

Subject	LAMP target	Sample (n)	DNA extraction	Sensitivity (%)	Specificity (%)	Reference test	Dis.	Country	Ref.
Human	kDNA	Blood (10)	Qiagen Mini prep	80.0	100	Microscopy	VL	Bangladesh	Takagi and colleagues 2009 [14]
Human	I8S rRNA	Blood (30) Skin biopsy (43)	Organic solvent	83.0 98.0	98.0 N/A	Microscopy qRT-PCR	VL CL	Sudan Suriname	Adams and colleagues 2010 [37]
Human	kDNA	Buffy- coat (75)	Qiagen Mini prep	90.7	100	Microscopy	VL	Bangladesh	Khan and colleagues 2012 [56]
Human	kDNA	Blood (55) BMA (15) Tissue biopsy (62)	Qiagen Mini prep	96.4 100 96.8	98.5 98.5 100	Microscopy qPCR	VL VL PKDL	India	Verma and colleagues 2013 [57]
Dog	cpb	Blood (75)	Wizard DNA kit	54.2	80.0	Microscopy	CanL	Tunisia	Chaouch and colleagues 2013 [72]
Human	kDNA	Blood (47)	Qiagen Mini prep	93.6	100	DAT	VL	Iran	Ghasemian and colleagues 2014 [59]
Human	18S rRNA	Blood (2) Saliva (2) Tissue biopsy (1) BMA (1)	Direct-Boil Qiagen Mini prep	N/A	N/A	Microscopy	VL CL CL VL	Thailand	Sirworarat and colleagues 2015 [41]
Dog	kDNA	Conjunct- Ional swab (111)	Boil-Spin	61.3	97.0	Microscopy	CanL	China	Gao and colleagues 2015 [73]
Human	kDNA	Tissue biopsies (31)	Qiagen Mini prep	82.6	100	Microscopy	CL	Sri Lanka	Kathalawala & Karunaweer, 2015 [45]
Human	18S rRNA	Tissue biopsy-FTA-card (122)	FTA purification reagent (Whatman)	N/A	N/A	Nested-PCR	CL	Peru	Nzelu and colleagues 2016 [9]
Human	kDNA	Blood (66) BMA (15) Tissue biopsy (67) Tissue biopsy (10)	Qiagen Mini prep	96.9 100 97.0 80.0	100 100 100 100	qPCR	VL VL PKDL CL	India	Verma and colleagues 2017 [44]
Human	18S rRNAand kDNA	Whole blood and Buffy-coat (185)	Boil-Spin and Qiagen Mini prep	97.6 100	99.0 99.0	Microscopy	VL	Sudan	Mukhtar and colleagues 2018 [20]
Human	18S rRNA	Tissue biopsy (2)	Direct-Boil	N/A	N/A	PCR	CL	Japan (Imported cases)	Imai and colleagues 2018 [42]
Human	18S rRNAand kDNA	Tissue biopsies (105) Whole blood PBMC Buffy-coat (50)	Qiagen Mini prep	95.0 92.3 88.5 92.3	86.0 100 95.8 95.8	Microscopy	CL VL VL VL	Colombia Ethiopia	Adams and colleagues 2018 [39]
Human	18S rRNAand kDNA	Tissue biopsy (274)	Qiagen Mini prep	92.2	94.1	Microscopy PCR	CL	Afghanistan	Vink et al. 2018 [46]
Human	kDNA	Blood (179) Blood (72)	Qiagen Mini prep Direct-blood-lysis	98.3 93.1	96.6 100	Microscopy rK39 qPCR	VL VL	India	Dixit and colleagues 2018 [58]

BMA, bone marrow aspirates; CanL, canine leishmaniasis; CL, cutaneous leishmaniasis; cpb, cysteine protease B; DAT, direct agglutination test; Dis, disease; FTA, Flinders Technology Associates; kDNA, kinetoplast DNA; LAMP, loop-mediated isothermal amplification; n, number of samples; N/A, not applicable; PBMC, peripheral blood mononuclear cells; PKDL, post kala-azar dermal leishmaniasis; qPCR, quantitative PCR; qRT-PCR, quantitative reverse-transcriptase polymerase chain reaction; Ref, references; rK39, recombinant antigen-based immunochromatography test; VL, visceral leishmaniasis

### VL and PKDL

VL is characterized by fever, weight loss, wasting, and splenomegaly and is fatal if left untreated. VL is caused by *L*. (*L*.) *infantum* (both in the New and Old World) or *L*. (*L*.) *donovani* (only in the Old World). The disease is prevalent in tropical regions, with more than 90% of total cases reported from India, Bangladesh, Sudan, Brazil, Ethiopia, and South Sudan [47]. Post kala-azar dermal leishmaniasis (PKDL) is a complication of VL, characterized by macular, maculopapular, and nodular lesions in a patient who has recovered from VL. Significantly, during interepidemic periods of VL, patients who developed PKDL are considered as potential reservoir hosts for the parasite [48]. Therefore, detection and identification of both VL and PKDL patients in endemic areas are of paramount importance to combat leishmaniasis.

Diagnosis of both VL and PKDL relies on microscopic examination of tissue smears; however, this method suffers from low sensitivity. For VL, microscopy has a sensitivity of 93%–99% for spleen aspirate, 53%–86% for bone marrow, and 53%–65% for lymph node aspirates [49], while for PKDL it has a sensitivity of 67%–100% for nodular lesions, 36%–69% for popular lesions, and 7%–33% for macular lesions [50]. Serological methods such as DAT, ELISA, and the rK39 strip test have high sensitivity but come with their own challenges—like an inability to discriminate between symptomatic and asymptomatic cases, cross-reactivity with other diseases, and inconsistent performance in HIV-VL co-infection cases—and are particularly not conclusive for PKDL diagnosis [51–52]. Like in the case of CL diagnosis, several PCR molecular-based tools have been successfully shown to have increased sensitivity and accuracy in VL and PKDL diagnosis using various templates [6, 35, 53]. Molecular tests are potentially important in the case of HIV-VL co-infection because of low antibody response in HIV-infected patients, which lowers the sensitivity of serological tests [54]. Among the three forms of leishmaniasis, the development of diagnostics for VL based on LAMP has gained more focus, and the Foundation for Innovative New Diagnostics (FIND) has also devoted its efforts towards reducing the burden of VL through innovative LAMP technique (http://www.finddiagnostics.org). Such interest on VL is not surprising because recent VL diagnostics modelling data indicate that early diagnosis and treatment of patients have the potential to immensely reduce the transmission of the disease in endemic areas [55]. Therefore, early detection of the infectious agent will require a simple and rapid specific test, which consequently makes LAMP an ideal test for VL.

Species-specific LAMP assays based on the kDNA gene have been largely established for VL and PKDL diagnosis in endemic areas (Table 1), as well as genus-specific LAMP for VL either based on the *Leishmania* 18S rRNA or ITS1 genes [28, 41], which were in-house assays. Loopamp *Leishmania* Detection kit (Eiken Chemical, Japan), a first *Leishmania* LAMP kit, which comes in a ready-to-use format with primers targeting both 18S rRNA and kDNA minicircles, specific to the *Leishmania* genus has also been evaluated for the diagnosis of VL [20]. There were no significant differences between the sensitivities or specificities of the LAMP kit test on whole blood and buffy-coat samples processed either by the simple boil and spin method or commercial QIAgen kits for VL in Sudan [20]. A similar study has shown that LAMP performed on DNA extracted from whole blood had good sensitivity compared to microscopy of highly invasive biopsy samples [39]. The diagnostic accuracy of LAMP testing for VL was reported as high (sensitivity 80%–100% and specificity 96%–100%) in all studies irrespective of the sample tested (whole blood, buffy coat) [14, 20, 37, 39, 44, 56–59] (Fig 3B and 3C). The amplification efficiency of LAMP in detection of *Leishmania* infection in multiple less or noninvasive DNA sources, such as saliva and peripheral blood [41], is valuable in reducing the probability of a false negative. In addition, LAMP may particularly be important for patients who are co-infected with human immunodeficiency virus (HIV). In patients suffering from both HIV and VL, LAMP was proven to have a good diagnostic efficacy with 100% (95% CI, 59%–100%) sensitivity and 100% (95% CI, 86%–100%) specificity [39, 41] (Fig 3D). Two studies provided diagnostic accuracy on LAMP testing for PKDL on tissue biopsies [44, 57]. The sensitivity and specificity were reported as high (97% and 100%, respectively) in both studies (Fig 3E). Furthermore, at VL and PKDL posttreatment stages, LAMP can potentially be utilized in the assessment of cure [44]; however, a study had previously revealed the inability of LAMP to amplify posttreatment patients when the number of parasites fell to extremely low levels, approximately 10 parasites/ml [37]. Variation in the LAMP detection limit or sensitivity may be related to the primers and the target region selected, the *Leishmania* species involved, and the DNA extraction methods employed. Taken collectively, LAMP has shown very good diagnostic performance for VL and PKDL—with high sensitivity and specificity similar to PCR methods—and has emerged as a promising POC test for screening at-risk populations, and HIV-VL diagnosis, as well as assessment of cure for VL and PKDL in endemic areas.

### Canine leishmaniasis (CanL)

Canine leishmaniasis (CanL) is an important zoonotic disease mainly caused by *L*. (*L*.) *infantum*, which is associated with the long history of companionship between dogs and humans, as well as with sand fly vectors. The disease exists in about 50 countries among the 98 countries where human leishmaniases are endemic, affecting mainly three foci: China, the Mediterranean basin, and Brazil [60]. Although *L*. (*L*.) *infantum* has been identified as the main aetiologic agent of CanL in the Old World [61], in the New World other species such as *L*. (*L*.) *chagasi* (*infantum*), *L*. (*L*.) *mexicana*, *L*. (*L*.) *amazonensis*, and *L*. (*V*.) *braziliensis* may be included as potential aetiologic agents [60, 62]. Canine infection with *Leishmania* has two effects: as a reservoir for human leishmaniasis and as a cause of a severe disease in dogs, which is usually fatal when left untreated [63]. Infected dogs play an important role in the transmission of leishmaniasis and as such represent a real threat to uninfected dogs and humans in endemic areas where sand fly vectors are present. Symptomatic dogs present clinical signs such as peripheral lymphadenopathy, weight loss, papular, nodular dermatitis, decreased appetite, lethargy, and splenomegaly [60, 64]. The diagnosis of CanL is complex due to its variable clinical manifestations and lack of symptoms during the early stage of the infection. Therefore, a reliable, accurate, and rapid diagnostic test is essential in early management of infected dogs and to prevent zoonotic transmission of the *Leishmania* parasite to humans in endemic areas. CanL diagnosis does not differ substantially from that in humans; several direct methods (culture of parasites and microscopy) and indirect methods (serological and molecular tests) are readily available for the diagnosis of CanL.

Parasitological and serological tests have limitations in the diagnosis of CanL, especially in asymptomatic dogs or during early infection [65]. Although parasitological diagnosis is the definitive methodology of detection and splenic aspirates are considered as the method of choice among lymph node and bone marrow for CanL diagnosis [66], CanL is frequently diagnosed through the detection of specific antibodies against *Leishmania* parasites, using serological techniques. However, serological tests (DAT, IFAT, ELISA) present some draw backs, such as cross-reaction with *Trypanosoma* parasites, species causing CL, and other hemoparasites; and false results in low titers or cases of immunological anergy [67]. Nevertheless, PCR with all its variants has had the greatest success due to its high sensitivity (89% to 100%) and specificity (95% to 100%) in the diagnosis of CanL [68–71].

Few studies have shown the utility of LAMP in the diagnosis of CanL (Table 1). A *L*. (*L*.) *infantum*-specific LAMP assay was developed successfully, targeting the cpb multicopy gene to detect parasites in the blood of 75 dogs using purified DNA [72]. The LAMP delivered 54.2% sensitivity and 80% specificity, and the results were comparable with nested-PCR but had lower sensitivity to IFAT (88.5%), which showed in contrast lower specificity (45%) [72]. A further study compared the performance of kDNA-based *L*. (*L*.) *infantum*-specific LAMP assay on conjunctival swab samples with conventional PCR, ELISA (serum), and microscopy (bone marrow) and found that LAMP detected 61.3% of infected dogs, which was similar to PCR (58.6%) and significantly higher than ELISA (40.5%) and microscopy (10.8%) [73]. However, the kDNA LAMP primer sets designed from a *L*. (*L*.) *infantum* strain isolated in China did not amplify strains from other countries and as such are not suitable for use in other endemic areas. Additionally, the study highlighted that the noninvasive sample collection resulted in a high uptake among dog owners, which is commendable for the control of CanL. These studies provide examples of how LAMP can be implemented in field detection and early management of CanL.

## Molecular xenomonitoring/surveillance potentials of LAMP for leishmaniasis

Molecular xenomonitoring (MX) is the screening of haematophagous insects for the presence of a pathogen’s genetic material (DNA/RNA) using molecular-based assays. Phlebotomine sand flies are the putative vectors of leishmaniases, and approximately 800 species have been recorded in five major genera: *Phlebotomus* and *Sergentomyia* in the Old World and *Lutzomyia*, *Brumptomyia*, and *Warileya* in the New World [74]. However, only species belonging to the genera *Phlebotomus* and *Lutzomyia* are the putative vectors of *Leishmania* [74, 75]. The spread of leishmaniasis largely depends on the distribution of sand fly vectors. Therefore, entomological monitoring of *Leishmania* infection in leishmaniasis endemic areas provides epidemiologic advantages for predicting the risk and expansion of the diseases, the estimation of which depends on the reliable identification of infected sand flies [27]. Additionally, estimation of infection rates in the vector could serve as an indicator of a change in transmission intensity and assessment of control programs [27].

**Fig 4:**
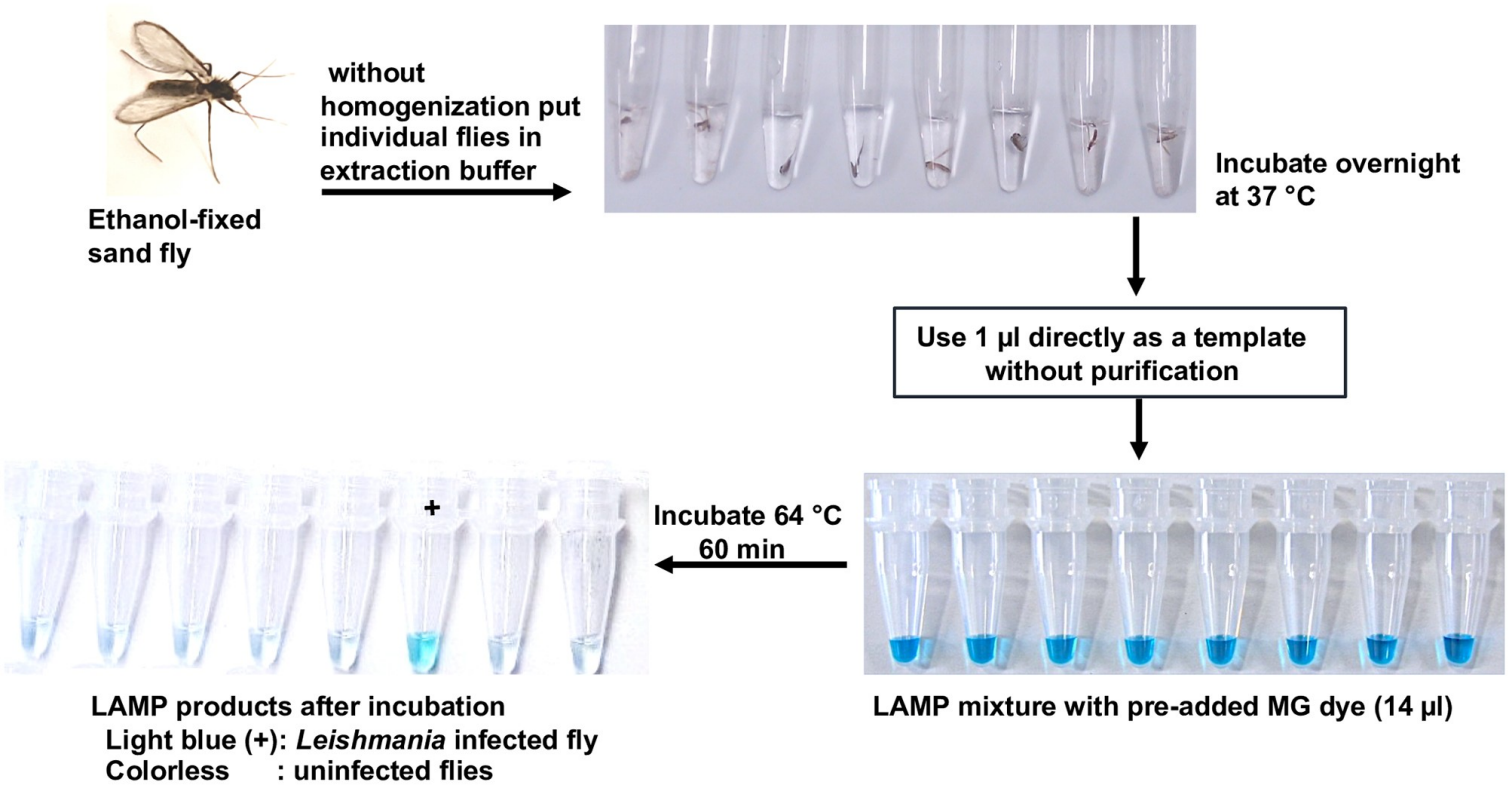
Graphic demonstration of colorimetric-MG-based LAMP assay for rapid mass-screening of individual sand flies for *Leishmania* infection.

Hitherto, detection of *Leishmania* parasites within individual sand flies relied largely on dissection and microscopic examination of individual flies, which is technically demanding, laborious, and time consuming—especially when large numbers of specimens must be examined due to the low *Leishmania* infection rate in sand flies (0.01%–1%), even in endemic areas [76]. To overcome these technical limitations, in the last three decades molecular approaches (PCR formats) have been increasingly employed in the detection of *Leishmania* DNA in individual or pooled sand flies [76, 77]. Of interest, LAMP has also been shown to be a good MX tool for generation of information on the distribution or expansion of leishmaniasis. In addition, from cost-effectiveness and field perspectives, LAMP offers some advantages over PCR as a usefulness method for surveillance and epidemiological studies of leishmaniasis in endemic areas. The first established LAMP for rapid mass-screening of individual sand flies for *Leishmania* infection was a generic-*Leishmania* 18S rRNA-based LAMP with preaddition of malachite green (MG) detection closed system, which can detect 0.01 parasites [27]. The field-based-MG-LAMP results using a crude sand fly template without DNA purification for *Leishmania* DNA detection (Fig 4) were comparable to classical microscopy and PCR. The LAMP was found to be a high-throughput screening tool for the detection of *Leishmania*-infected *Lutzomyia* sand fly species (8 out of 397 field-caught flies) in the endemic areas of Ecuador [27]. Another study validated the established LAMP for individual screening of 150 wild-caught sand flies from endemic areas of Iran and revealed that the 18S rRNA-based-LAMP detected 10 *Leishmania* DNA positive sand flies (*Sergentomyia baghdadis*, *S*. *sintoni*, and *Phlebotomus papatasi*) [78]. A similar study also experimentally validated the potential field applicability of the MG-LAMP for detection of *L*. *martiniquensis* DNA in sand flies in Thailand; however, no *Leishmania* DNA was detected by LAMP and PCR when applied to 380 field-caught flies, probably due to low infection rate in the study area [79]. Additionally, the performance of the MG-LAMP was further validated using purified DNA from sand fly pools and was found to be of potential use for the entomological surveillance of CL in Colombia with 100% sensitivity and 96.8% specificity [80]. Indeed, these reported studies have shown the potential usefulness of LAMP for mass-screening of sand flies in the Old and New World. Importantly, LAMP allows for an immediate real-time assessment of the presence of *Leishmania* parasites in endemic foci, which demonstrates the possibility of its integration as a simple and cost-effective molecular tool for monitoring or surveillance of infections and identification of the vector species.

## LAMP amplicon end-point closed detection system

A wide range of approaches are available for the qualitative detection of LAMP amplicons. The amplified products can be detected visually using multiple parameters, including turbidity, fluorescence, and color with the naked eyes and/or UV light (Table 2). Following agarose gel electrophoresis, an open detection system is usually used as a confirmatory analysis of the LAMP products that appear as cauliflower-like structures with multiple loops. The presence of turbidity (white color precipitate) in sample from byproducts of amplification has been used as the indicator of a positive *Leishmania* DNA LAMP reaction, while absence of turbidity indicated a negative LAMP reaction [43, 56]. However, turbidity is challenging to discern and is unstable over time. As an alternative to turbidity, intercalating fluorescent dyes were used for direct detection of the *Leishmania* DNA LAMP positive samples such as calcein [73], SYBR Green I [57, 78], fluorescent detection reagents (FDR) (Eiken) [37, 43], which are technically inconvenient due to its requirement for a UV illuminator for test result discrimination, and can bind nonspecifically to any dsDNA, even to the primer-dimers leading to an incorrect interpretation of results [81]. In addition, fluorescence-based assays require specialized equipment such as the LED illuminators [21] to enable the readout of the fluorescence dyes or the use of real-time fluorimetry [21, 28]. The use of specialized equipment for the visualization of the LAMP results reduces the versatility of the LAMP and tends to increase the overall cost of the LAMP assay, which might hinder its use in resource-limited countries. On the other hand, despite the reported good sensitivity, SYBR Green I could inhibit the LAMP reaction if added before isothermal incubation. Therefore, the dye must be introduced post-LAMP reaction, requiring the LAMP tube to be opened or postamplification handling (open detection system), which is a contamination risk and usually leads to false-positive results. Recently, studies have shown the use of SYBR Green I placed on the inner side of the cap of reaction tube in a closed-LAMP assay; however, the assay requires an additional step of brief spin after LAMP reaction for amplicon discrimination [44, 58]. The detection of amplicon with preaddition of FDR eliminates the openings of tubes and reduces contamination problems; however, FDR has low detection sensitivity [82] and is costly [37]. Colorimetric-LAMP detection dyes, like hydroxy naphthol blue (HNB) and MG, have also been utilized for the naked eye visualization of positive LAMP products in closed systems. The addition of HNB and MG dyes in the LAMP reaction tube prior amplification eliminates the opening of tubes and completely avoids contamination problems [9, 27]. HNB dye changes from violet to a sky blue color [82] and requires the operator to distinguish between positive and negative color results, which can be ambiguous. In contrast, MG signal recognition is highly sensitive, the system gives a clear-cut difference between positives (light blue color) and negatives (colorless) based on naked eye visualization, and interpretation by an independent observer is not required [9, 27, 41, 80]. MG-LAMP products are stable and thus can be kept for record purposes. Several studies have shown the reliability, robust sensitivity, and wide applicability of MG-based-LAMP closed detection system for *Leishmania* and other purposes. MG has been used for detection of *Leishmania* DNA in crude *Lutzomyia* sand fly extract in Ecuador [27]; patient’s tissue spotted on FTA-card in Peru [9]; blood, saliva, and tissue biopsies in Thailand [41]; and direct smears and sand flies in Colombia [80]. Taken together, the incorporation of a closed-LAMP assay offer new prospects for improved detection of infections, as well as positioning LAMP as a quick, one-step, POC molecular diagnostic and xenomonitoring tool for leishmaniasis and other diseases.

**Table 2:** Summary of methods used in LAMP amplicon end-point detection in previous studies.

Detection parameter	Open/ closed system	Evaluation of results	Equipment for end-point detection	Remarks	References
Turbidity	Closed	Turbid—positive Clear—negative	Turbidimeter or none	Not always easy to interpret	Khan and colleagues 2012 [56]; Mikita and colleagues 2014 [43]
Gel-electrophoresis	Open	Ladder-like bands—positive No band—negative	UV transilluminator	Prone to contamination	Nzelu and colleagues 2014 [27]; Gao and colleagues 2015 [73]
Calcein	Closed	Green—positive Orange—negative	UV lamp	Inconvenient due to dangerous UV illuminator	Gao and colleagues 2015 [73]
SYBR Green I	Open or closed	Green—positive Orange—negative	UV light or none	Inhibits LAMP reaction and prone to contamination when added postreaction	Verma and colleagues 2013, 2017 [44, 57]; Ghodrati and colleagues 2017 [78]; Dixit and colleagues 2018 [58]
FDR (Eiken)	Closed	Fluorescent green—positive Pale brown—negative	UV light	Expensive	Adams and colleagues 2010 [37]; Mikita and colleagues 2014 [43]
Hydroxy naphthol blue	Closed	Sky blue—positive Violet—negative	Light box (optional) or none	Ambiguous to discern, requires operator to distinguish results	Goto and colleagues 2009 [82]
Malachite green	Closed	Light blue—positive Colorless—negative	None	Easy to discern results by the naked eye; stable and can be kept for record purposes	Nzelu and colleagues 2014 [27], 2016 [9]; Sriworarat and colleagues 2015 [41]; León and colleagues 2018 [80]

FDR, Fluorescent detection reagent; LAMP, loop-mediated isothermal amplification: UV, ultra-violet

## Cost and hands-on time of the *Leishmania* LAMP test

Besides technical feasibility and performance, the major issue related to the large-scale and routine implementation of molecular diagnostics is its cost-effectiveness. LAMP has proven to be applicable to field detection, potentially eliminating the need for expensive DNA purification kits, thermal cyclers, and gel electrophoresis. The reported costs of *Leishmania* LAMP range from less than US$1–$3.50 per test [37, 43], compared to US$12 for qPCR, US$2.50 for PCR-restriction fragment length polymorphism (PCR-RFLP) [34, 37], US$1.5–2.5 for DAT, around US$1 for rk39-based immunochromatographic-RDT [83], and less than US$1 for microscopy per test. Some studies have suggested that the use of a very cheap colorimetric dye like MG [9, 80] for LAMP amplicon visualization, instead of Eiken fluorescent detection reagent [37], and heat treated samples as a template DNA source [41–43, 58] may further reduce the cost per test. A study indicated that a malaria LAMP assay using heat treated blood cost between US$0.40 and US$0.70, which is lower than currently available RDT’s [13]. Although *Leishmania*-RDT has a cost similar to microscopy when used at reference centers/clinics, LAMP appears to be more appropriate and cost effective during CL seasons or outbreaks, when its capacity to process several tests at a time can be maximized [84]. Moreover, lowering the cost of LAMP will broaden its application, especially in resources-limited countries where leishmaniasis is endemic.

Rapid turnaround time is equally an important aspect of any test, and patients and clinicians require a test that can produce a result rapidly for prompt treatment. The hands-on time to result for closed-LAMP is 30–60 mins [9, 20, 44], compared to 3–6 hours for most PCR assays and 10–20 mins for rK39-RDT [83]. LAMP, being much faster than PCR assays, falls in the category of a high performance result oriented NAAT, which will support leishmaniasis elimination initiatives.

## Concluding remarks and future directions

The development and application of LAMP technique in the diagnosis of leishmaniasis cases in endemic areas is in line with the recent global trend in seeking rapid, POC tests for the control of infectious diseases. LAMP appears to be an ideal diagnostic test suitable for neglected and forgotten tropical diseases in the world, including leishmaniasis. Furthermore, it meets the guidelines laid down by the World Health Organization (WHO) that diagnostics for developing countries should be ASSURED: Affordable, Sensitive, Specific, User-friendly, Rapid and robust, Equipment-free, and Deliverable to end users [85]. In this review, we compiled the diagnostic performance and recent advances employing the LAMP assay to detect *Leishmania* in human CL, VL, PKDL, CanL, and sand flies. Based on the current review and discussion, the following points should be considered. 1) Making diagnostic techniques patient-centric (in-clinic and in the field) was the main aim behind the advent of LAMP as a POC test; therefore, LAMP quantitative diagnostic methods that were developed are not applicable as POC tests because they require relatively costly equipment and personnel. Quantitative assays are good for special purposes, such as research. 2) The sensitivity and specificity of LAMP largely depends on the primer sets employed; hence, care must be taken when designing primers. Although it is challenging to choose a correct and proper target for amplification (either a highly conserved region or a target site that is species-specific), it is imperative to ensure that the assay amplifies the predicted target and is specific, which may require some preliminary experimental optimization before final primer selection. While the Primer Explorer software can be employed to design primer sets, successful primers can be manually designed even when preferred target sites are not selected by the software. 3) One of the most attractive features of LAMP is the ability to overcome potential inhibitors in unpurified templates. The use of boiled clinical samples [41–43], direct-blood lysis [58], and crude sand fly extract [27] reduces time, cost, and the requirement for extensive laboratory infrastructure. The stability of the target DNA in crude sand fly extract stored at −20°C for months/years has been reported [27] but has not been reported for boiled supernatant (boil and spin or Direct Boil-LAMP). Hence, it is important to develop buffers that can stabilize DNA in the supernatant in order to ensure subsequent amplification consistency before it can be relied upon as template. 4) High risk of carry-over contamination, which often leads to false-positive results in supposedly negative controls, is a major challenge of LAMP. Amplicons are usually stable, and as such, unintended carry-over contamination may occur. It is recommended to adopt a closed end-point detection system in order to avoid postamplification contamination. 5) The Loopamp *Leishmania* Detection Kit—which is in a ready-to-use format, has an additional advantage of dried reagents including *Leishmania* genus-specific primers, and has shelf life of one year if stored between 1 and 30°C (Eiken)—is a further advancement of LAMP. Additionally, an electricity-free stable heat block based on exothermic chemical reactions and phase-change material has been demonstrated for amplification reaction [86], and the use of rechargeable solar batteries as alternative energy-source has also been proposed [81]. However, the development of a consensus standardized integrated system from simple sample collection and preservation, template preparation, and amplification platform to closed detection unit for end-point use will be essential for field and in-clinic testing even in the most remote rural endemic areas. 6) It is important to highlight that LAMP positivity should always be interpreted in combination with clinic pathological evaluations. Focusing on the aforementioned points will undoubtedly improve the application of LAMP in the diagnosis of leishmaniasis. Importantly, LAMP has come to stay as a potential rapid POC test for diagnosis and entomological monitoring of *Leishmania* infection in endemic areas.

**Key Learning Points**

- Leishmaniasis remains one of the world’s most neglected and poverty-related tropical diseases, and early detection of *Leishmania* will require a simple and rapid diagnostic test for timely treatment of patients.
- A closed LAMP diagnostic tool with high specificity, sensitivity, rapidity, and simplicity provides an effective point-of-care test applicable in *Leishmania* endemic and nonendemic areas.
- LAMP tests are potentially important in the diagnosis of HIV-VL co-infection cases because of low antibody response in HIV-infected patients, which lowers the sensitivity of serological tests.
- A rapid and reliable diagnostic test is important for the management of *Leishmania*-infected dogs to prevent zoonotic transmission of the parasite in endemic areas.
- LAMP allows for an immediate real-time assessment of the presence of *Leishmania* parasites in endemic foci, and monitoring of *Leishmania* infection in sand flies can provide epidemiologic data for predicting the risk and expansion of the disease.

**Top Five Papers**

1. Adams ER, Schoone G, Versteeg I, Gomes MA, Diro E, Mori Y, et al. Development and evaluation of a novel loop mediated isothermal amplification assay for the diagnosis of cutaneous and visceral leishmaniasis. J Clin Microbiol. 2018; 56 (7): e00386–18.
2. Gao C, Ding D, Wang J, Steverding D, Wang X, Yang Y, et al. Development of a LAMP assay for detection of *Leishmania infantum* infection in dogs using conjunctival swab samples. Parasit Vectors. 2015; 8: 370.
3. Notomi T, Okayama H, Masubuchi H, Yonekawa T, Watanabe K, Amino N. Loop-mediated isothermal amplification of DNA. Nucleic Acids Res. 2000; 28 (12): E63.
4. Nzelu CO, Gomez EA, Caceres AG, Sakurai T, Martini-Robles L, Uezato H, et al. Development of a loop-mediated isothermal amplification method for rapid mass-screening of sand flies for *Leishmania* infection. Acta Trop. 2014; 132: 1–6.
5. Verma S, Singh R, Sharma V, Bumb RA, Negi NS, Ramesh V, et al. Development of a rapid loop-mediated isothermal amplification assay for diagnosis and assessment of cure of *Leishmania* infection. BMC Infect Dis. 2017; 17 (1), 223.

## References

[1] Hotez PJ, Pecoul B, Raijai S, Boehme C, Aksoy S, Malecela M, et al Eliminating the neglected tropical diseases: translational science and new technologies. PLoS Negl Trop Dis. 2016; 10(3):e0003895 10.1371/journal.pntd.0003895 26934395 PMC4774924

[2] Alvar J, Vélez ID, Bern C, Herrero M, Desjeux P, Cano J, et al Leishmaniasis worldwide and global estimates of its incidence. PLoS ONE. 2012; 7 (5): e35671 10.1371/journal.pone.0035671 22693548 PMC3365071

[3] McGwire BS, Satoskar AR. Leishmaniasis: clinical syndromes and treatment. Q J Med. 2014; 107:7–14. 10.1093/qjmed/hct116 PMC3869292 23744570

[4] Boelaert M, Verdonck K, Menten J, Sunyoto T, van Griensven J, Chappuis F, et al Rapid tests for the diagnosis of visceral leishmaniasis in patients with suspected disease. Cochrane Database Syst Rev. 2014; 6: CD009135. 10.1002/14651858.CD009135.pub2 PMC4468926 24947503

[5] Cunningham J, Hasker E, Das P, El Safi S, Goto H, Mondal D, et al A global comparative evaluation of commercial immunochromatographic rapid diagnostic tests for visceral leishmaniasis. Clin Infect Dis. 2012; 55 (10): 1312–1319. 10.1093/cid/cis716 22942208 PMC3478143

[6] de Ruiter CM, van der Veer C, Leeflang MM, Deborggraeve S, Lucas C, Adams ER. Molecular tools for diagnosis of visceral leishmaniasis: systematic review and meta-analysis of diagnostic test accuracy. J Clin Microbiol. 2014; 52(9): 3147–3155. 10.1128/JCM.00372-14 24829226 PMC4313130

[7] de Vries HJC, Reedijk SH, Schallig HDFH. Cutaneous leishmaniasis: recent developments in diagnosis and management. Am J Clin Dermatol. 2015; 16(2):99–109. 10.1007/s40257-015-0114-z 25687688 PMC4363483

[8] Kato H, Caceres AG, Mimori T, Ishimaru Y, Sayed ASM, Fujita M, et al Use of FTA cards for direct sampling of patients’ lesions in the ecological study of cutaneous leishmaniasis. J Clin Microbiol. 2010; 48 (10): 3661–3665. 10.1128/JCM.00498-10 20720027 PMC2953078

[9] Nzelu CO, Caceres AG, Guerrero-Quincho S, Tineo-Villafuerte E, Rodriquez-Delfin L, Mimori T, et al A rapid molecular diagnosis of cutaneous leishmaniasis by colorimetric malachite green-loop-mediated isothermal amplification (LAMP) combined with an FTA card as a direct sampling tool. Acta Trop. 2016; 153: 116–119. 10.1016/j.actatropica.2015.10.013 26516109

[10] Notomi T, Okayama H, Masubuchi H, Yonekawa T, Watanabe K, Amino N, et al Loop-mediated isothermal amplification of DNA. Nucleic Acids Res. 2000; 28 (12): E63 10.1093/nar/28.12.e63 10871386 PMC102748

[11] Notomi T, Mori Y, Tomita N, Kanda H. Loop-mediated isothermal amplification (LAMP): principle, features, and future prospects. J Microbiol. 2015; 53 (1): 1–5. 10.1007/s12275-015-4656-9 25557475

[12] Iwamoto T, Sonobe T, Hayashi K. Loop-mediated isothermal amplification for direct detection of mycobacterium tuberculosis complex *M*. *avium* and *M*. *intracellulare* in sputum samples. J Clin Microbiol. 2003; 41 (6): 2616–2622. 10.1128/JCM.41.6.2616-2622.2003 12791888 PMC156570

[13] Poon LL, Wong BW, Ma EH, Chan KH, Chow LM, Abeyewickreme W, et al Sensitive and inexpensive molecular test for falciparum malaria: detecting *Plasmodium falciparum* DNA directly from heat-treated blood by loop-mediated isothermal amplification. Clin Chem. 2006; 52 (2), 303–306. 10.1373/clinchem.2005.057901 16339303

[14] Takagi H, Itoh M, Islam MZ, Razzaque A, Ekram ARM, Hashiguchi Y, et al Sensitive, specific and rapid detection of *Leishmania donovani* DNA by loop-mediated isothermal amplification. Am J Trop Med Hyg. 2009; 81(4): 578–582. 10.4269/ajtmh.2009.09-0145 19815869

[15] Bao H, Zhao Y, Wang Y, Xu X, Shi J, Zeng X, et al Development of a reverse transcription loop-mediated isothermal amplification method for the rapid detection of subtype H7N9 avian influenza virus. Biomed Res Int. 2014; 525064 10.1155/2014/525064 24689044 PMC3933526

[16] Njiru ZK, Mikosza AS, Armstrong T, Enyaru JC, Ndung’u JM, Thompson AR. Loop-mediated isothermal amplification (LAMP) method for rapid detection of *Trypanosoma brucei rhodesiense*. PLoS Negl Trop Dis. 2008; 2 (1): e147 10.1371/journal.pntd.0000147 18253475 PMC2238707

[17] Mitarai S, Okumura M, Toyota E, Yoshiyama T, Aono A, Sejimo A, et al Evaluation of a simple loop-mediated isothermal amplification test kit for the diagnosis of tuberculosis. Int J Tuberc Lung Dis. 2011; 15 (9):1211–1217. 10.5588/ijtld.10.0629 21943848

[18] Hopkins H, Gonález IJ, Polley SD, Angutoko P, Ategeka J, Asilmwe C, et al Highly sensitive detection of malaria parasitemia in a malaria-endemic setting: performance of a new loop-mediated isothermal amplification kit in a remote clinic in Uganda. J Infect Dis. 2013; 208 (4): 645–652. 10.1093/infdis/jit184 23633405 PMC3719898

[19] Polley SD, Gonzá lez IJ, Mohamed D, Daly R, Bowers K, Watson J, et al Clinical evaluation of a loop-mediated amplification kit for diagnosis of imported malaria. J Infect Dis. 2013; 208 (4): 637–644. 10.1093/infdis/jit183 23633403 PMC3719897

[20] Mukhtar M, Ali SS, Boshara SA, Albertini A, Monnerat S, Bessell P, et al Sensitive and less invasive confirmatory diagnosis of visceral leishmaniasis in Sudan using loop-mediated isothermal amplification (LAMP). PLoS Negl Trop Dis. 2018; 12 (2): e0006264 10.1371/journal.pntd.0006264 29444079 PMC5828521

[21] Ibarra-Meneses A, Cruz I, Chicharri C, Sánchez C, Biéler S, Broger T, et al Evaluation of fluorimetry and direct visualization to interpret results of a loop-mediated isothermal amplification kit to detect *Leishmania* DNA. Parasit Vectors. 2018; 17: 11 (1): 250 10.1186/s13071-018-2836-2 29665825 PMC5905109

[22] WHO. The use of loop-mediated isothermal amplification (TB-LAMP) for the diagnosis of pulmonary tuberculosis: policy guidance. 2016. WHO/HTM/TB/2016.07. http://apps.who.int/iris/bitstream/10665/249154/1/9789241511186-eng.pdf. [cited 2017 February 15]. 27606385

[23] Rypien C, Chow B, Chan WW, Church DL, Pillai DR. Detection of *Plasmodium* infection by the illumigene malaria assay compared to reference microscopy and real-time PCR. J Clin Microbiol. 2017; 55 (10): 3037–3045. 10.1128/JCM.00806-17 28768730 PMC5625390

[24] Piepenburg O, Williams CH, Stemle DL, Armes NA. DNA detection using recombination proteins. PLoS Biol. 2006; 4: e204 10.1371/journal.pbio.0040204 16756388 PMC1475771

[25] Gill P, Ghaemi A. Nucleic acid isothermal amplification technologies: a review. Nucleosides Nucleotides Nucleic Acids. 2008; 27 (3): 224–243. 10.1080/15257770701845204 18260008

[26] Nagamine K, Hase T, Notomi T. Accelerated reaction by loop-mediated isothermal amplification using loop primers. Mol Cell Probes. 2002; 16 (3): 223–229. 12144774 10.1006/mcpr.2002.0415

[27] Nzelu CO, Gomez EA, Caceres AG, Sakurai T, Martini-Robles L, Uezato H, et al Development of a loop-mediated isothermal amplification method for rapid mass-screening of sand flies for *Leishmania* infection. Acta Trop. 2014; 132: 1–6. 10.1016/j.actatropica.2013.12.016 24388795

[28] Abbasi I, Kirstein OD, Hailu A, Warburg A. Optimization of loop-mediated isothermal amplification (LAMP) assays for the detection of *Leishmania* DNA in human blood samples. Acta Trop. 2016;162: 20–26 10.1016/j.actatropica.2016.06.009 27288706 PMC4987123

[29] Desjeux P. Leishmaniasis: current situation and new perspectives. Comp Immunol Microbiol Infect Dis. 2004; 27 (5): 305–318. 10.1016/j.cimid.2004.03.004 15225981

[30] WHO. Control of the leishmaniasis: report of a meeting of the WHO Expert Committee on the control of leishmaniasis. World Health Organ Tech Rep Ser 949, Geneva. 2010.

[31] Hashiguchi Y, Velez LN, Villegas NV, Mimori T, Gomes EAL, Kato H. Leishmaniasis in Ecuador: comprehensive review and current status. Acta Trop. 2017;166: 299–315. 10.1016/j.actatropica.2016.11.039 27919688

[32] Hashiguchi Y, Gomes EL, Kato H, Martini LR, Velez LN, Uezato H. Diffuse and disseminated cutaneous leishmaniasis: -cases experienced in Ecuador and a brief review. Trop Med Health. 2016; 44: 2. 27398061 10.1186/s41182-016-0002-0 PMC4934146

[33] Gradoni L. Conn’s Current Therapy, Infectious Diseases. Elsevier, Philadelphia, PA, 2016; pp.134–136.

[34] Reithinger R, Dujardin JC. Molecular diagnosis of leishmaniasis: current status and future applications. J Clin Microbiol. 2007; 45 (1): 21–25. 10.1128/JCM.02029-06 17093038 PMC1828971

[35] Akhoundi M, Downing T, Votýpka J, Kuhls K, Lukeš J, Cannet A, et al *Leishmania* infections: molecular targets and diagnosis. Mol Aspects Med. 2017; 57:1–29. 10.1016/j.mam.2016.11.012 28159546

[36] Adams ER, Gomes MA, Scheske L, Rios R, Marquez R, Cossio A, et al Sensitive diagnosis of cutaneous leishmaniasis by lesion swab sampling coupled to qPCR. Parasitology. 2014; 141 (14):1891–1897. 10.1017/S0031182014001280 25111885 PMC4654403

[37] Adams ER, Schoone GJ, Ageed AF, Safi SE, Schallig HD. Development of a reverse transcriptase loop-mediated isothermal amplification (LAMP) assay for the sensitive detection of *Leishmania* parasites in clinical samples. Am J Trop Med Hyg. 2010; 82(4): 591–596. 10.4269/ajtmh.2010.09-0369 20348505 PMC2844582

[38] Yurchenko VY, Merzlyak EM, Kolesnikov AA, Martinkina LP, Vengerov YY. Structure of *Leishmania* minicircle kinetoplast DNA classes. J Clin Microbiol. 1999; 37 (5), 1656–1657. 10328690 10.1128/jcm.37.5.1656-1657.1999 PMC84871

[39] Adams ER, Schoone G, Versteeg I, Gomes MA, Diro E, Mori Y, et al Development and evaluation of a novel loop mediated isothermal amplification assay for the diagnosis of cutaneous and visceral leishmaniasis. J Clin Microbiol. 2018; 56 (7): e00386–18. 10.1128/JCM.00386-18 29695527 PMC6018344

[40] Chaouch M, Aoun K, Ben Othman S, Ben Abid M, Ben Sghaier I, Bouratbine A, et al Development and assessment of *Leishmania major*- and *Leishmania tropica*-specific loop-mediated isothermal amplification assays for the diagnosis of cutaneous leishmaniasis in Tunisia. Am J Trop Med Hyg. 2019;19–0097. 10.4269/ajtmh.19-0097 PMC6609195 31094311

[41] Sriworarat C, Phumee A, Mungthin M, Leelayoova S, Siriyasatien P. Development of loop- mediated isothermal amplification (LAMP) for simple detection of *Leishmania* infection. Parasit Vectors. 2015; 8: 591 10.1186/s13071-015-1202-x 26577333 PMC4650110

[42] Imai K, Tarumoto N, Amo K, Takahashi M, Sakamoto N, Kosaka A, et al Non-invasive diagnosis of cutaneous leishmaniasis by the direct boil loop-mediated isothermal amplification method and MinION^™^ nanopore sequencing. Parasitol Int. 2018; 67 (1): 34–37. 10.1016/j.parint.2017.03.001 28288843

[43] Mikita K, Maeda T, Yoshikawa S, Ono T, Miyahira Y, Kawana A. The direct boil-LAMP method: a simple and rapid diagnostic method for cutaneous leishmaniasis. Parasitol. Int. 2014; 63(6), 785–789. 10.1016/j.parint.2014.07.007 25086375

[44] Verma S, Singh R, Sharma V, Bumb RA, Negi NS, Ramesh V, et al Development of a rapid loop-mediated isothermal amplification assay for diagnosis and assessment of cure of *Leishmania* infection. BMC Infect Dis. 2017; 17 (1), 223 10.1186/s12879-017-2318-8 28335752 PMC5363003

[45] Kothalawala HS, Karunaweera ND. Loop-mediated isothermal amplification assay as a sensitive diagnostic tool for *Leishmania donovani* infections in Sri Lanka. Ceylon Med J. 2016; 61(2): 68–70. 10.4038/cmj.v61i2.8286 27423747 PMC6206497

[46] Vink MMT, Nahzat SM, Rahimi H, Buhler C, Ahmadi BA, Nader M, et al Evaluation of point-of-care tests for cutaneous leishmaniasis diagnosis in Kabul Afghanistan. EBioMedicine. 2018; 37: 453–460. 10.1016/j.ebiom.2018.10.063 30396855 PMC6286266

[47] WHO. Leishmaniasis in high-burden countries: an epidemiological update based on data reported in 2014. Wkly Epidemiol Rec. 2016; 91 (22): 285–296 27263128

[48] Desjeux P, Ghosh RS, Dhalaria P, Strub-Wourgaft N, Zijlstra EE. Report of the post kala- azar dermal leishmaniasis (PKDL) consortium meeting, New Delhi India, 27–29. Parasit Vectors. 2013; 6:196. 23819611 10.1186/1756-3305-6-196 PMC3733610

[49] Siddig M, Ghalib H, Shillington DC, Petersen EA. Visceral leishmaniasis in the Sudan: comparative parasitological methods of diagnosis. Trans R Soc Trop Med Hyg. 1988; 82: (1): 66–68. 3176153

[50] Salotra P, Singh R. Challenges in the diagnosis of post kala-azar dermal leishmaniasis. Indian J Med Res. 2006; 123 (3): 295–310. 16778312

[51] Singh R, Raju BVS, Jain RK, Salotra P. Potential of direct agglutination test based on promastigote and amastigote antigens for serodiagnosis of post-kala-azar dermal leishmaniasis. Clin Diagn Lab Immunol. 2005; 12 (10): 1191–1194. 10.1128/CDLI.12.10.1191-1194.2005 16210482 PMC1247836

[52] Srivastava P. Dayama A. Mehrotra S. Sundar S. Diagnosis of visceral leishmaniasis. Trans R Soc Med Hyg. 2011; 105 (1): 1–6. 10.1016/j.trstmh.2010.09.006 PMC2999003 21074233

[53] Sreenivas G, Anasari NA, Kataria J, Salotra P. Nested PCR assay for detection of *Leishmania donovani* in slit aspirates from post-kala-azar dermal leishmaniasis lesion. J Clin Microbial. 2004; 42 (4): 1777–1778. 10.1128/JCM.42.4.1777-1778.2004 PMC387583 15071047

[54] Cota GF, de sousa MR, Demarqui FN, Rabello A. The diagnostic accuracy of serologic and molecular methods for detecting visceral leishmaniasis in HIV infected patients: meta-analysis. PLoS Negl Trop Dis. 2012; 5 (5):e1665. 10.1371/journal.pntd.0001665 PMC3362615 22666514

[55] Medley GF, Hollingsworth TD, Olliaro PL, Adams ER. Health-seeking behavior, diagnostics and transmission dynamics in the control of visceral leishmaniasis in the Indian subcontinent. Nature. 2015; 528(7580): S102–108. 10.1038/nature16042 26633763

[56] Khan MG, Bhaskar KR, Salam MA, Akther T, Pluschke G, Mondai D. Diagnostic accuracy of loop-mediated isothermal amplification (LAMP) for detection of *Leishmania* DNA in buffy coat from visceral leishmaniasis patients. Parasit Vectors. 2012; 5:280 10.1186/1756-3305-5-280 23206441 PMC3545740

[57] Verma S, Avishek K, Sharma V, Negi NS, Ramesh V, Salotra P. Application of loop-mediated isothermal amplification assay for the sensitive and rapid diagnosis of visceral leishmaniasis and post-kala-azar dermal leishmaniasis. Diagn Microbiol Infect Dis. 2013; 75 (4): 390–395. 10.1016/j.diagmicrobio.2013.01.011 23433714

[58] Dixit KK, Verma S, Singh OP, Singh D, Singh AP, Guota R, et al Validation of SYBR Green I based closed tube loop mediated isothermal amplification (LAMP) assay and simplified direct-blood-lysis (DBL)-LAMP assay for diagnosis of visceral leishmaniasis (VL). PLoS Negl Trop Dis. 2018; 12 (11): e0006922 10.1371/journal.pntd.0006922 30439953 PMC6264900

[59] Ghasemian M, Gharavi MJ, Akhlaghi L, Mohebali M, Meamar AR, Aryan E, et al Development and assessment of loop-mediated isothermal amplification (LAMP) assay for the diagnosis of human visceral leishmaniasis in Iran. Iranian J Parasitol. 2014; 9 (1): 50–59. PMC4289880 25642260

[60] Alvar J, Cañavate C, Molina R, Moreno J, Nieto J. Canine leishmaniasis. Adv Parasitol. 2004; 57:1–88. 10.1016/S0065-308X(04)57001-X 15504537

[61] Gramiccia M, Gradoni L. The current status of zoonotic leishmaniases and approaches to disease control. Int J Parasitol. 2005; 35 (11–12): 1169–1180. 10.1016/j.ijpara.2005.07.001 16162348

[62] Lainson R, Shaw JJ. Leishmaniasis in the new world In: Collier L. Balows A., Sussman M. (Eds.), Topley & Wilson’s Microbiology and Microbial Infections, vol. 5, 10th ed Parasitology, Arnold, London 2005; pp. 313–349.

[63] Reguera RM, Moran M, Perez-Pertejo Y, Garcia-Estrada C, Balana-Fouce R. Current status on prevention and treatment of canine leishmaniasis. Vet Parasitol. 2016; 227: 98–114. 10.1016/j.vetpar.2016.07.011 27523945

[64] Noli C, Saridomichelakis MN. An update on the diagnosis and treatment of canine leishmaniasis caused by *Leishmania infantum* (syn. *L*. *chagasi*). Vet J. 2014; 202 (3): 425–435. 10.1016/j.tvjl.2014.09.002 25266647

[65] Ferreira ED, de Lana M, Carneiro M, Reis AB, Paes DV, da Silva ES, et al Comparison of serological assays for the diagnosis of canine visceral leishmaniasis in animals presenting different clinical manifestations. Vet Parasitol. 2007; 146 (3–4): 235–241. 10.1016/j.vetpar.2007.02.015 17403582

[66] Barrouin-Melo SM, Larangeira DF, Trigo J, Aguiar PH, dos-Santos WL, Pontes-de-Carvalho L. Comparison between splenic and lymph node aspirations as sampling methods for the parasitological detection of *Leishmania chagasi* infection in dogs. Mem. Inst. Oswaldo Cruz. 2004; 99 (2): 195–197. 10.1590/s0074-02762004000200014 15250475

[67] Lopes EG, Seva AP, Ferreira F, Nunes CM, Keid LB, Hiramoto RM, et al Serological and molecular diagnostic tests for canine leishmaniasis in Brazilian endemic area: one out of five seronegative dogs are infected. Epidemiol Infect. 2017; 145 (12): 2436–2444. 10.1017/S0950268817001443 28726597 PMC9148790

[68] Lachaud L, Chabbert E, Dubessay P, Dereure J, Lamothe J, Dedet JP, et al Value of two PCR methods for the diagnosis of canine visceral leishmaniasis and the detection of asymptomatic carriers. Parasitology. 2002; 125(3): 197–207. 12358417 10.1017/s0031182002002081

[69] Moreira M, Luvizotto MCR, Garcia JF, Corbett C, Laurenti M. Comparison of parasitological, immunological and molecular methods for the diagnosis of leishmaniasis in dogs with different clinical signs. Vet Parasitol. 2007; 145 (3–4): 245–252. 10.1016/j.vetpar.2006.12.012 17257764

[70] Carson C, Quinnell RJ, Holden J, Garcez LM, Deborggraeve S, Courtenay O. Comparison of *Leishmania* OligoC-Test PCR with conventional and real-time PCR for diagnosis of canine *Leishmania* infection. J Clin Microbiol. 2010; 48 (9): 3325–3330. 10.1128/JCM.02331-09 20631112 PMC2937666

[71] Mohammadiha A, Mohebali M, Haghighi A, Mahdian R, Abadi AR, Zarei Z, et al Comparison of real-time PCR and conventional PCR with two DNA targets for detection of *Leishmania* (*Leishmania*) *infantum* infection in human and dog blood sample. Exp Parasitol. 2013; 133 (1): 89–94. 10.1016/j.exppara.2012.10.017 23159412

[72] Chaouch M, Mhadhbi M, Adams ER, Schoone GJ, Limam S, Gharbi Z, et al Development and evaluation of a loop-mediated isothermal amplification (LAMP) assay for rapid detection of *Leishmania infantum* in canine leishmaniasis based on cysteine protease b gene. Vet Parasitol. 2013; 198 (1–2): 78–84. 10.1016/j.vetpar.2013.07.038 23972768

[73] Gao C, Ding D, Wang J, Steverding D, Wang X, Yang Y, et al Development of a LAMP assay for detection of *Leishmania infantum* infection in dogs using conjunctival swab samples. Parasit Vectors. 2015; 8: 370 10.1186/s13071-015-0991-2 26169060 PMC4501202

[74] Munstermann LE. Phlebotomine sand flies, the Psychodidae In: Marquardt WC, Black WC, Freier JE, Hagedorn HH, Hemingway J, et al, editor. Biology of Disease Vectors, Second ed Elsevier, San Diego, CA 2004: pp. 141–151.

[75] Kato H, Gomez EA, Cáceres AG, Uezato H, Mimori T, Hashiguchi Y. Molecular epidemiology for vector research on leishmaniasis. Int J Environ Res Public Health. 2010; 7 (3): 814–826. 10.3390/ijerph7030814 20617005 PMC2872317

[76] Kato H, Uezato H, Katakura K, Calvopiña M, Marco JD, Barroso PA, et al Detection and identification of *Leishmania* species within naturally infected sand flies in the Andean areas of Ecuador by a polymerase chain reaction. Am J Trop Med Hyg. 2005; 72 (1): 87–93. 15728872

[77] Nzelu CO, Kato H, Puplampu N, Desewu K, Odoom S, Wilsom MD, et al First detection of *Leishmania tropica* DNA and *Trypanosoma* species in *Sergentomyia* sand flies (Diptera: Psychodidae) from an outbreak area of cutaneous leishmaniasis in Ghana. PLoS Negl Trop Dis. 2014; 8: e2630 10.1371/journal.pntd.0002630 24516676 PMC3916256

[78] Ghodrati M, Spotin A, Hazratian T, Mahami-Oskouei M, Bordbar A, Ebrahimi S, et al Diagnosis accuracy of loop-mediated isothermal amplification assay as a field molecular tool for rapid mass screening of old world *Leishmania* infections in sand flies and in vitro culture. Iran J Parasitol. 2017; 12(4): 506–515. 29317875 PMC5756300

[79] Tiwanathagorn S, Kato H, Yeewa R, Muengpan A, Polseela R, Leelayoova S. Comparison of LAMP and PCR for molecular mass screening of sand flies for *Leishmania martiniquensis* infection. Mem Inst Oswaldo Cruz. 2017; 112 (2), 100–107. 10.1590/0074-02760160254 28177044 PMC5293119

[80] León CM, Muñoz M, Tabares JH, Hernandez C, Florez C, Ayala MS, et al Analytical performance of a loop-mediated isothermal amplification assay for *Leishmania* DNA detection in sand flies and direct smears of patients with cutaneous leishmaniasis. Am J Trop Med Hyg. 2018; 98 (5): 1325–1331. 10.4269/ajtmh.17-0808 29532767 PMC5953379

[81] Njiru ZK. Loop-mediated isothermal amplification technology: towards point of care diagnostics. PLoS Negl Trop Dis. 2012; 6 (6): e1572 10.1371/journal.pntd.0001572 22745836 PMC3383729

[82] Goto M, Honda E, Ogura A, Nomoto A, Hanaki K. Colorimetric detection of loop-mediated isothermal amplification reaction by using hydroxyl naphthol blue. Biotechniques. 2009; 46 (3): 167–172. 10.2144/000113072 19317660

[83] Singh OP, Sundar S. Developments in diagnosis of visceral leishmaniasis in the elimination era. J Parasitol Res. 2015: 239469 10.1155/2015/239469 26843964 PMC4710934

[84] Aerts C, Vink M, Pashtoon SJ, Nahzat S, Picado A, Cruz I, et al Cost effectiveness of new diagnostic tools for cutaneous leishmaniasis in Afghanistan. Appl Health Econ Health Policy. 2019; 17 (2): 213–230. 10.1007/s40258-018-0449-8 30465319 PMC6439180

[85] Mabey D, Peeling RW, Ustianowski A, Perkins MD. Diagnostics for the developing world. Nat Rev Microbiol. 2004; 2: 231–240. 10.1038/nrmicro841 15083158

[86] LaBarre P, Hawkins KR, Gerlach J, Wilmoth J, Beddoe A, Singleton J, et al A simple, inexpensive device for nucleic acid amplification without electricity–towards instrument-free molecular diagnostics in low-resource settings. PLoS ONE. 2011; 6 (5): e19738 10.1371/journal.pone.0019738 21573065 PMC3090398

